# Associations between COVID-19 outcomes and asthmatic patients with inhaled corticosteroid

**DOI:** 10.3389/fphar.2023.1204297

**Published:** 2023-11-01

**Authors:** Su-Boon Yong, Shuo-Yan Gau, Chia-Jung Li, Chih-Wei Tseng, Shiow-Ing Wang, James Cheng-Chung Wei

**Affiliations:** ^1^ Department of Allergy and Immunology, China Medical University Children’s Hospital, Taichung, Taiwan; ^2^ Research Center for Allergy, Immunology, and Microbiome (A.I.M.), China Medical University Hospital, Taichung, Taiwan; ^3^ School of Medicine, Chung Shan Medical University, Taichung, Taiwan; ^4^ Department of Obstetrics and Gynecology, Kaohsiung Veterans General Hospital, Kaohsiung, Taiwan; ^5^ Division of Allergy, Immunology and Rheumatology, Department of Internal Medicine, Taichung Veterans General Hospital, Taichung, Taiwan; ^6^ Department of Public Health, Collage of Medicine, National Cheng Kung University, Tainan, Taiwan; ^7^ Institute of Medicine, Chung Shan Medical University, Taichung, Taiwan; ^8^ Center for Health Data Science, Department of Medical Research, Chung Shan Medical University Hospital, Taichung, Taiwan; ^9^ Department of Allergy, Immunology and Rheumatology, Chung Shan Medical University Hospital, Taichung, Taiwan; ^10^ Graduate Institute of Integrated Medicine, China Medical University, Taichung, Taiwan

**Keywords:** COVID-19, epidemiology, cohort, TriNetX database, asthma

## Abstract

**Background:** The impact of inhaled corticosteroid (ICS) in the interaction between asthma, COVID-19 and COVID-19 associated outcomes remain largely unknown. The objective of this study is to investigate the risk of COVID-19 and its related outcomes in patients with asthma using and not using inhaled corticosteroid (ICS).

**Methods:** We used the TriNetX Network, a global federated network that comprises 55 healthcare organizations (HCO) in the United States, to conduct a retrospective cohort study. Patients with a diagnosis of asthma with and without ICS between January 2020 and December 2022 were included. Propensity score matching was used to match the case cohorts. Risks of COVID-19 incidence and medical utilizations were evaluated.

**Results:** Out of 64,587 asthmatic patients with ICS and without ICS, asthmatic patients with ICS had a higher incidence of COVID-19 (Hazard ratio, HR: 1.383, 95% confidence interval, CI: 1.330–1.437). On the contrary, asthmatic patients with ICS revealed a significantly lower risk of hospitalization (HR: 0.664, 95% CI: 0.647–0.681), emergency department visits (HR: 0.774, 95% CI: 0.755–0.793), and mortality (HR:0.834, 95% CI:0.740–0.939). In addition, subgroup or sensitivity analyses were also conducted to examine the result of different vaccination status, disease severity, or COVID-19 virus variants.

**Conclusion:** For asthmatic patients using ICS, risk of COVID-19 was significantly higher than non-users. The observed association could provide potential guidance for primary care physicians regarding the risk of COVID-19 in asthmatic patients.

## Highlights

• Inhaled corticosteroid (ICS) utilization is associated with increased risk of incident COVID-19.
• Among asthma patients, ICS users had lower risk of hospitalization and emergency room visit than non-users.

## Introduction

Asthma is one of the most common chronic diseases worldwide, affecting approximately 272 million people of all ages (Skevaki et al., 2020; Thompson et al., 2021). Uncontrolled asthma can have direct and indirect costs that are 10 times higher than those of controlled patients (Skevaki et al., 2020; Eger and Bel, 2021). Viral infections and virus-induced exacerbations (i.e., rhinovirus) can affect asthma control (Skevaki et al., 2020; Eger and Bel, 2021).

COVID-19 is a pandemic with more than 580 million confirmed cases and more than 6.4 million deaths worldwide as of August 2022 (Papadopoulos et al., 2020; Skevaki et al., 2020; Eger and Bel, 2021; Green et al., 2021; Thompson et al., 2021). Airborne infections lead to severe pulmonary outcomes, including acute respiratory distress syndrome and interstitial pneumonia (Convertino et al., 2020).

Patients often die of respiratory failure. As COVID-19 patients are infected through the respiratory tract, it was speculated that patients with asthma had a potentially heightened risk of acquiring SARS-CoV-2 infection as well as worse COVID-19 outcomes (Papadopoulos et al., 2020; Skevaki et al., 2020; Eger and Bel, 2021; Green et al., 2021).

The severity of COVID-19 is often linked to changes in the status of white blood cell, particularly the presence of lymphocytopenia, with indications that cytokine storm conditions are associated with disease severity. In patients with severe COVID-19 symptoms, the serum TNF alpha and IL-6 were reported to be massively elevated (Convertino et al., 2020). Inhaled corticosteroids (ICS), which are important drugs for treating asthma, act directly on the respiratory tract (Skevaki et al., 2020; Eger and Bel, 2021; Green et al., 2021). Concerns were raised regarding the use of ICS and COVID-19 infection. Choi et al., using national cohort data in Korea, showed that asthma led to poor COVID-19 outcomes, but the medications and the severity of asthma based on the medications prescribed were not independent predictors for poor outcomes (Choi et al., 2021).

However, according to a study by Aveyard et al., asthma does not appear to be associated with an increased risk of severe COVID-19. In contrast, chronic obstructive lung disease and interstitial lung disease are linked to a 50% higher risk of severe COVID-19. It's important to note that while these increased risks exist, they are relatively small when compared to factors like gender and diabetes. These findings should be considered within the broader context of overall mortality risk, potentially helping to alleviate anxiety among individuals with respiratory conditions. Additionally, the relationship between the use of inhaled steroids and the risk of severe COVID-19 remains uncertain (Aveyard et al., 2021). Nonetheless, the use of ICS was associated with a modestly increased risk of severe COVID-19 (Aveyard et al., 2021). In contrast, a study by Bloom et al. (2021) indicates that the use of inhaled corticosteroids within 2 weeks of admission may improve survival for patients aged 50 years and older with asthma, but not for those with chronic pulmonary disease. Patients admitted to the hospital with COVID-19 often have underlying respiratory conditions, and irrespective of symptom severity and comorbidities, individuals with asthma were more likely to receive critical care, whereas those with chronic pulmonary disease were less likely to do so compared to those without respiratory conditions.

Conflicting results regarding whether a diagnosis of asthma increases the risk of severe COVID-19 and whether the use of ICS has an impact on the prognosis is unclear. The purpose of this study was to evaluate medical data on asthmatic patients with and without ICS use to determine any associations with the prognosis for COVID-19. To our knowledge, this is the first study to use TriNetX data to examine the association between COVID-19 and asthmatic patients with ICS and without ICS. We aimed to study the risks of incident COVID-19 risk and medical utilization in asthma patients with and without ICS.

## Materials and methods

### Study design and data source

This was a retrospective cohort study. The data used in the present study was aggregated from TriNetX, the world’s largest, living ecosystem of real-world data and evidence for the life sciences and healthcare. It contains de-identified electronic health records of more than 250 million persons from more than 120 global healthcare organizations (HCOs) from countries in North and South America, EMEA, and Asia-Pacific, including Japa, providing up-to-the month real-time data. TriNetX employs a standardized framework to ensure data quality, which encompasses three primary categories of quality metrics: conformance, completeness, and plausibility (Kahn et al., 2016). It has been utilized for the execution of numerous studies of high quality (Paljarvi et al., 2022; Taquet et al., 2022). More detailed information about TriNetX is available on its web site: https://trinetx.com/. The variables which can be captured from TriNetX include demographics, diagnoses (represented by International Classification of Diseases, Tenth Revision, Clinical Modification, ICD-10-CM codes), procedures (coded in The International Classification of Diseases, Tenth Revision, Procedure Coding System, ICD-10-PCS or Current Procedural Terminology, CPT), medication (coded in Veterans Affairs (VA) National Formulary or Anatomical Therapeutic Chemical (ATC) Classification System), laboratory measures (coded in Logical Observation Identifiers Names and Codes, LOINC), genomics (coded in Human Genome Variation Society, HGVS), and healthcare utilization.

Data and analysis were done in June, 2023. We used the US Collaborative Network, the subnet of TriNetX platform to perform the related analysis. This network includes 56 HCOs. Due to our study objective, the duration of the study was limited to the period between 1 January 2020 and 31 December 2022.

### Study subjects

**FIGURE 1:**
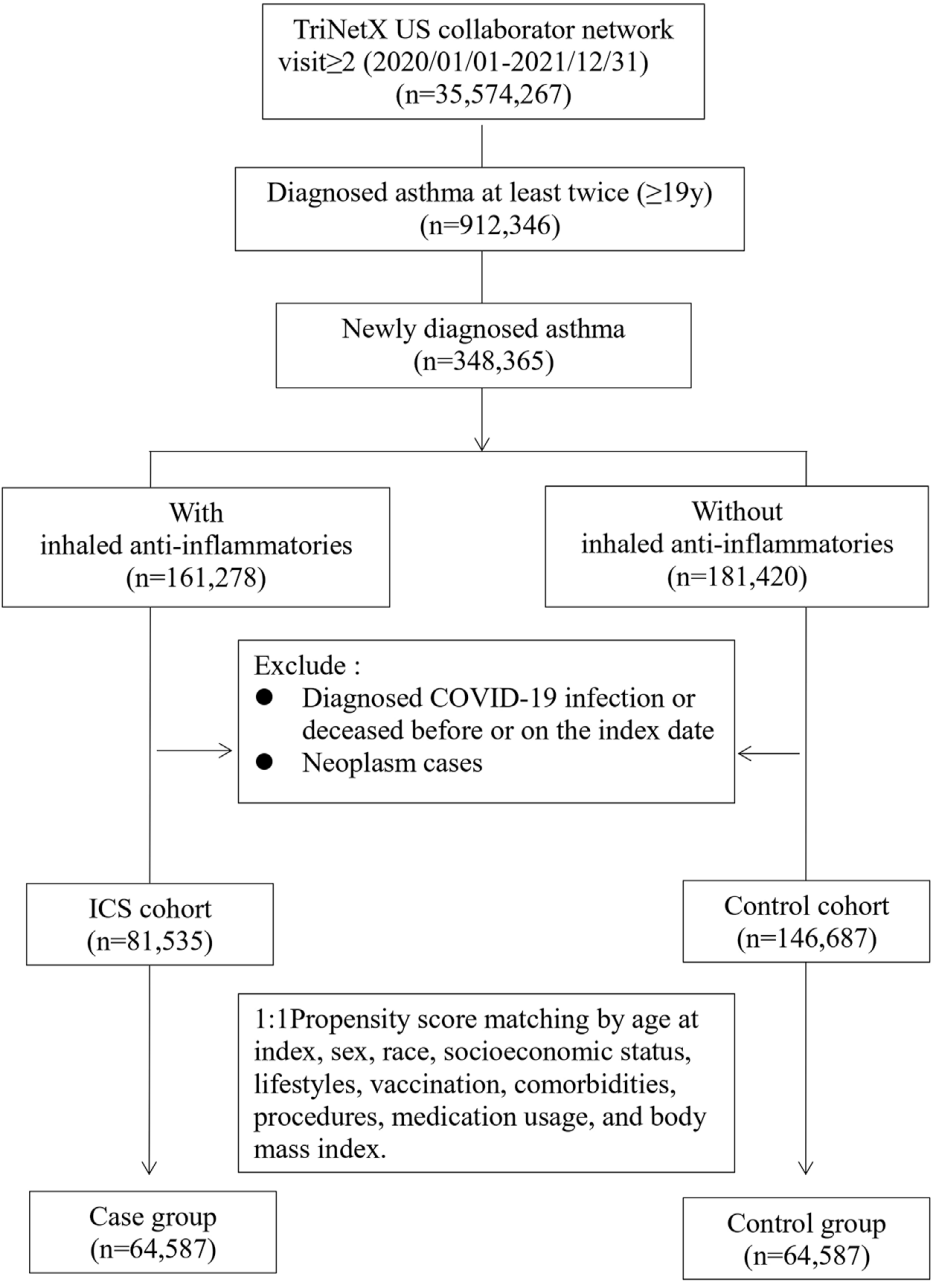
Flow chart of selection.

Study subjects included newly diagnosed adult asthma (ICD-10-CM code J45) patients (≥19 years old) enrolled in the TriNetX US database between 1 January 2020 and 31 December 2022. The study subjects were then divided into two cohorts according to whether the patients had been prescribed inhaled anti-inflammatory medicine (VA code RE101), which included mometasone (ATC code R01AD09), flunisolide (R01AD08), beclomethasone (R03BA01), budesonide (R03BA02), fluticasone (R03BA05), and ciclesonide (R03BA08). The index date for the ICS cohort was set as the date when the inhaled anti-inflammatory medicine was first prescribed, whereas the index date for the control cohort was established according to the date of the initial asthma diagnosis. Selection process were presented in Figure 1. In both cohorts, patients who had been diagnosed with COVID-19 (ICD-10 code: U07.1, U07.2, U09, Z86.16, J12.82 or related RNA confirmation, detailed in Supplementary Table S1) prior to the index date were excluded. We also excluded those who deceased before or on the index date. Neoplasm patients were also excluded.

### Definition of covariates

The following covariate factors (within 1 year prior to the index date) were incorporated into the present study to reduce confounding effects.

#### Demographics

Age at index was used; sex was coded as female or male; race was encoded as White, Black or African American, Asian, and American Indian or Native Hawaiian; and social economic status was encoded as a proxy code (ICD 10 code Z59 Problems related to housing and economic circumstances, and Z56 Problems related to employment and unemployment).

#### Lifestyles

Nicotine dependence (ICD 10 code F17) and tobacco use (ICD 10 code Z72.0) were encoded as proxy codes for smoking. Alcohol-related disorders (ICD 10 code F10) were used as a proxy for alcohol drinking status.

#### Comorbidities

All comorbidities were coded as presence or absence, i.e., dichotomous variables, and were coded as ICD-10 codes. The comorbidities used in the present study included gastro-esophageal reflux disease without esophagitis (ICD 10 code K21.9), liver disease (K70-K77), essential hypertension (I10), diabetes mellitus (E08-E13), disorders of lipoprotein metabolism (E78), systemic lupus erythematosus (M32), Sjogren syndrome (M35.0), chronic kidney disease (N18), gastritis and duodenitis (K29), depression (F32), ischemic heart disease (I20-I25), and overweight and obesity (E66).

#### Procedure/medications

COVID-19 vaccination status was considered an important factor that may be related to outcomes, and thus the present study incorporated COVID-19 vaccine data into the analysis. Medications were divided into user or non-user based on the prescription information, and included adrenergics, inhalants (ATC code R03A), adrenergics for systemic use (ATC code R03C), other systemic drugs for obstructive airway diseases (ATC code R03D), vasoprotective corticosteroids (ATC code C05AA), and corticosteroids for systemic use, plain (ATC code H02A).

#### Laboratory data

Laboratory results were also included in the analysis to explore the baseline characteristics between the two cohorts, and included body mass index (kg/m^2^) and immunoglobulin E (IgE,[IU]/mL).

### Outcomes

The outcomes of interest in the present study were

1. The incidence of COVID-19, defined by ICD 10 codes (U07.1: COVID-19 virus identified, U07.2: COVID-19, virus not identified, U09: Post COVID-19 condition, J12.82: Pneumonia due to COVID-19, Z86.16: Personal history of COVID-19) or confirmed by laboratory RNA testing (Supplementary Table S1).
2. Medical utilization, which included hospitalization (CPT code 1013659, 1013699,1013729, or visit encoded as inpatient), emergency room visit (CPT code 1013711), critical/intensive care (CPT code 1013729), and mechanical ventilation (CPT code 31500, 1015098, 5A1935Z, 5A1945Z, 5A1955Z, 0BH17EZ, 0BH18EZ, 0BH13EZ, 1022227, or ICD-9 procedure code 39.65 (Extracorporeal membrane oxygenation, ECMO).
3. All-cause mortality, defined by vital status record (deceased) encoding in TriNetX. The mortality information in the TriNetX database was gathered from Social Security Administration and published obituary records.

Both cohorts were followed up between 1 day after the index date to 365 days.

### Statistical analyses

We used the built-in capability of TriNetX to generate propensity scores and performed 1: 1 matching using greedy nearest neighbor matching with a caliper of 0.1 pooled standard deviations of the two groups. In addition, to examine the effect of adjusting for different factors on the results, this study aggregated the results of four models, namely, 1) model 1: before matching, 2) model 2: matching with age at index, sex, race, 3) model 3: matching with age at index, sex, race, social economic status (SES), lifestyles, vaccination and body mass index (BMI), and 4) model 4: matching with age at index, sex, race, SES, lifestyles, comorbidities, vaccination, medication usage, and body mass index (BMI). Comparisons between two groups before and after matching were explored with a standardized mean difference. If the standardized mean difference was lower than 0.1, it was considered a good match. Kaplan-Meier analysis was used to estimate the probability of the outcome of interest including mortalities, medical utilization, including hospitalization and emergency room visit, critical/intensive care, and mechanical ventilation. The hazard ratio (HR) and its associated confidence intervals (CI), together with the test for proportionality were calculated using R’s Survival package v3.2–3. The proportional hazard assumption was tested using the generalized Schoenfeld approach. The TriNetX platform also runs a suite of tests, comparing the outcome from independent, industry-standard methods, to verify that all counts, rates, and statistics are calculated correctly as outlined in https://support.trinetx.com/hc/en-us/articles/360053133594-How-does-TriNetX-test-for-proportionality-on-a-hazard-ratio-. Log-Rank test results indicated whether the survival curves were different between groups, and were done within TriNetX. Subgroup analyses based on COVID-19 vaccination status (*vaccinated with COVID-19 related vaccines before index date, never vaccinated with a related vaccine,* detailed in Supplementary Table S2) and disease severity (*severe: inpatient* or used emergency department services, critical care services *within 1* *month on or after the index date, non-severe: never hospitalization, emergency room visit, or critical care services within 1* *month on or after the index date*) were performed to explore the differences among those groups. Sensitivity analyses were performed to illustrate the consistency of results among different virus (Alpha, Delta, Omicron) time wave.

## Results

### Characteristics of study subjects

The basic characteristics of the subjects before and after matching are shown in Table 1. Before matching, the two cohorts were significantly different in age at index, lifestyles, comorbidities, and medication usage. After matching, the differences between two cohorts were within the acceptable range (standardized mean difference <0.1). After propensity score matching, a total of 64,587 cases who were prescribed with an inhaled anti-inflammatory medication and a control cohort with the same number of people who had never been prescribed an inhaled anti-inflammatory medication were identified in our study.

**TABLE 1:** Baseline characteristics of study subjects (before and after propensity score matching).

Variables	Before matching			After matching^a^		
	ICS cohort (n = 102,805)	Control cohort (n = 126,031)	Std diff	ICS cohort (n = 64,587)	Control cohort (n = 64,587)	Std diff
**Age at index**						
Mean ± SD	47.3 ± 17.7	42.3 ± 17.3	**0.284**	45.7 ± 17.6	45.8 ± 17.8	0.010
**Sex**						
Female	68,871 (67.0)	84,033 (66.7)	0.007	43,048 (66.7)	43,101 (66.7)	0.002
Male	33,895 (33.0)	41,951 (33.3)	0.007	21,516 (33.3)	21,456 (33.2)	0.002
**Race, n (%)**						
White	63,326 (61.6)	74,396 (59.0)	0.053	39,547 (61.2)	40,225 (62.3)	0.022
Black or African American	17,585 (17.1)	26,198 (20.8)	0.094	11,285 (17.5)	10,471 (16.2)	0.034
Asian	2,829 (02.8)	2,863 (02.3)	0.031	1,729 (02.7)	1,776 (02.7)	0.004
American Indian or Alaska Native	492 (00.5)	632 (00.5)	0.003	329 (00.5)	315 (00.5)	0.003
Native Hawaiian or Other Pacific Islander	156 (00.2)	212 (00.2)	0.004	98 (00.2)	87 (00.1)	0.005
**Social economic status**						
Housing/economic circumstances problem	512 (00.5)	426 (00.3)	0.025	258 (00.4)	259 (00.4)	<0.001
Employment or unemployment problems	214 (00.2)	129 (00.1)	0.027	105 (00.2)	104 (00.2)	<0.001
**Lifestyles**						
Nicotine dependence	7,171 (07.0)	5,626 (04.5)	**0.108**	3,642 (05.6)	3,654 (05.7)	0.001
Alcohol related disorders	1,586 (01.5)	1,280 (01.0)	0.047	810 (01.3)	827 (01.3)	0.002
Tobacco use	1,932 (01.9)	1,021 (00.8)	0.093	836 (01.3)	813 (01.3)	0.003
**Comorbidities**						
Gastro-esophageal reflux disease without esophagitis	13,363 (13.0)	5,958 (04.7)	**0.294**	5,486 (08.5)	5,570 (08.6)	0.005
Diseases of liver	2,651 (02.6)	1,639 (01.3)	0.093	1,272 (02.0)	1,256 (01.9)	0.002
Essential (primary) hypertension	22,336 (21.7)	13,548 (10.7)	**0.301**	10,696 (16.6)	10,960 (17.0)	0.011
Diabetes mellitus (DM)	9,770 (09.5)	6,618 (05.3)	**0.163**	4,899 (07.6)	4,885 (07.6)	0.001
Disorders of lipoprotein metabolism	17,870 (17.4)	9,946 (07.9)	**0.289**	8,324 (12.9)	8,476 (13.1)	0.007
Systemic lupus erythematosus (SLE)	529 (00.5)	354 (00.3)	0.037	276 (00.4)	254 (00.4)	0.005
Sjögren syndrome	376 (00.4)	198 (00.2)	0.041	174 (00.3)	172 (00.3)	0.001
Chronic kidney disease (CKD)	3,060 (03.0)	1,902 (01.5)	0.099	1,444 (02.2)	1,414 (02.2)	0.003
Gastritis and duodenitis	1,302 (01.3)	825 (00.7)	0.063	649 (01.0)	648 (01.0)	<0.001
Depressive episode	9,309 (09.1)	5,699 (04.5)	**0.181**	4,360 (06.8)	4,527 (07.0)	0.010
Ischemic heart diseases	5,122 (05.0)	2,834 (02.2)	**0.147**	2,264 (03.5)	2,264 (03.5)	<0.001
Overweight and obesity	13,429 (13.1)	7,830 (06.2)	**0.234**	6,229 (09.6)	6,324 (09.8)	0.005
**Procedure**						
COVID-19 vaccination						
BNT	2,291 (02.2)	1,415 (01.1)	0.086	1,097 (01.7)	1,226 (01.9)	0.015
Moderna	425 (00.4)	234 (00.2)	0.042	183 (00.3)	195 (00.3)	0.003
Janssen	45 (00.0)	61 (00.0)	0.002	30 (00.0)	28 (00.0)	0.001
**Medication**						
Adrenergics, inhalants	52,347 (50.9)	17,023 (13.5)	**0.874**	17,182 (26.6)	17,023 (26.4)	0.006
Adrenergics for systemic use	48,994 (47.7)	15,759 (12.5)	**0.830**	15,891 (24.6)	15,724 (24.3)	0.006
Other systemic drugs for obstructive airway diseases	15,076 (14.7)	3,375 (02.7)	**0.436**	3,843 (06.0)	3,374 (05.2)	0.032
Corticosteroids for systemic use, plain	33,650 (32.7)	14,911 (11.8)	**0.519**	13,073 (20.2)	13,336 (20.6)	0.010
**Lab**						
Body mass index, Mean ± SD, kg/m^2^	30.9 ± 7.4	30.2 ± 7.6	0.092	30.6 ± 7.4	30.7 ± 7.6	0.006
Total IgE in Serum, Mean ± SD, [IU]/mL	313.8 ± 545.1	165.3 ± 363.1	**0.321**	271.7 ± 570.3	192.7 ± 430.4	**0.156**

Note: ICS, inhaled corticosteroid; Std diff, Standardized difference; COVID-19, coronavirus disease 2019; BNT, Pfizer–BioNTech COVID-19, vaccine; SD, standard deviation.

If the patient had a value of less than or equal to 10, results show the count as 10.

Bold font represents a standardized difference was more than 0.1.

^a^ Propensity score matching was performed on age at index, sex, race, social economic status, lifestyles, vaccination, comorbidities, medication usage, and body mass index.

#### COVID-19 incidence

**FIGURE 2:**
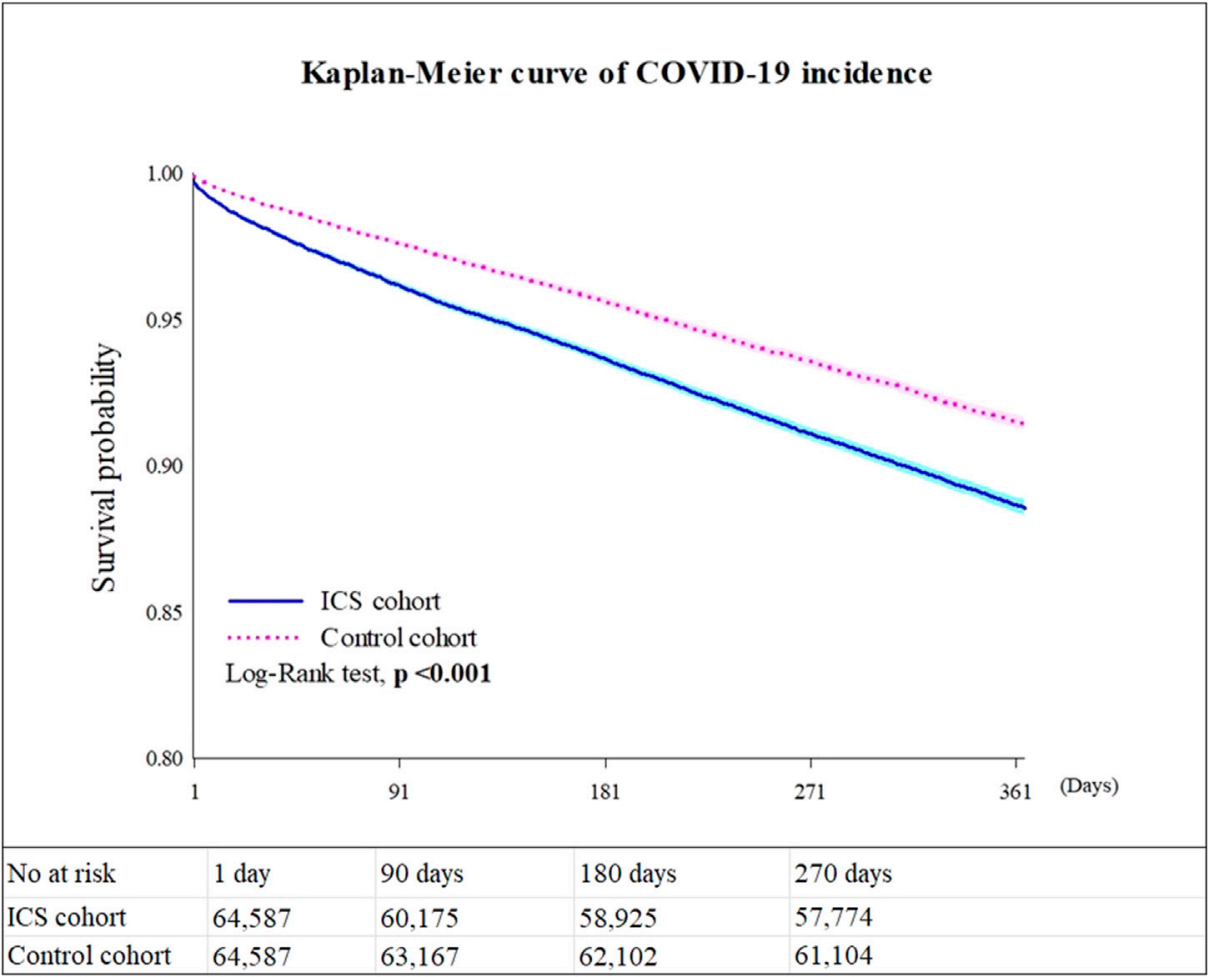
Kaplan-Meier survival curves for COVID-19 incidence. *Legends:* Values presented in Figure 2 are based on Model 4.

Table 2 shows the risk of outcomes between the ICS cohort and the control cohort in different models. Compared to the control cohort, patients who were prescribed an inhaled anti-inflammatory medication had a higher incidence of COVID-19 (Hazard ratio, HR: 1.383, 95% confidence interval, CI: 1.330–1.437, proportionality <0.001) in all models. The Kaplan-Meier curves of COVID-19 incidence are shown in Figure 2. The log-rank test revealed a significant difference between the ICS users and the control cohort (*p* < 0.001). Due to the results violate the proportional hazard assumption, we further present the results from different study period to explore the results over time (Supplementary Table S3).

**TABLE 2:** Risk of outcomes in ICS cohort compared to control cohort.

Outcome (ICS vs. control cohort)	Hazard ratio (95% CI)			
	Model 1 (crude)	Model 2	Model 3	Model 4
COVID-19 incidence	1.504 (1.461–1.548)*	1.554 (1.505–1.604)*	1.521 (1.473–1.571)*	1.383 (1.330–1.437)*
Medical utilization				
Hospitalization	0.735 (0.721–0.749)*	0.739 (0.724–0.754)	0.705 (0.691–0.720)	0.664 (0.647–0.681)
Emergency room visit	0.772 (0.758–0.786)*	0.847 (0.831–0.864)*	0.807 (0.791–0.824)*	0.774 (0.755–0.793)*
Critical/intensive care	1.174 (1.110–1.241)	1.089 (1.025–1.156)	1.013 (0.952–1.078)	0.953 (0.881–1.031)
Mechanical ventilation	1.316 (1.213–1.427)	1.202 (1.102–1.310)	1.114 (1.019–1.218)	1.048 (0.937–1.172)
All-cause mortality				
Deceased	1.192 (1.092–1.302)	0.970 (0.884–1.065)	0.900 (0.817–0.990)	0.834 (0.740–0.939)

ICS, inhaled corticosteroid; COVID, coronavirus disease; CI, confidence interval.

Model 1: before matching.

Model 2: matching with age at index, sex, race.

Model 3: matching with age at index, sex, race, social economic status (SES), lifestyles, vaccination and body mass index.

Model 4: matching with age at index, sex, race, SES, lifestyles, vaccination, comorbidities, medication usage, and body mass index.

*proportionality <0.001.

#### Medical utilization

Subjects who were prescribed an inhaled anti-inflammatory medication had significantly decreased risks of medical utilization, including hospitalization (HR: 0.664, 95% CI: 0.647–0.681), and emergency department visits (HR: 0.774, 95% CI: 0.755–0.793), (Table 2).

#### Mortality

The ICS cohort exhibited a significant lower mortality risk than control cohort (HR: 0.834, 95%CI: 0.740–0.939) (Table 2).

### Subgroup analyses

#### COVID-19 vaccination

**FIGURE 3:**
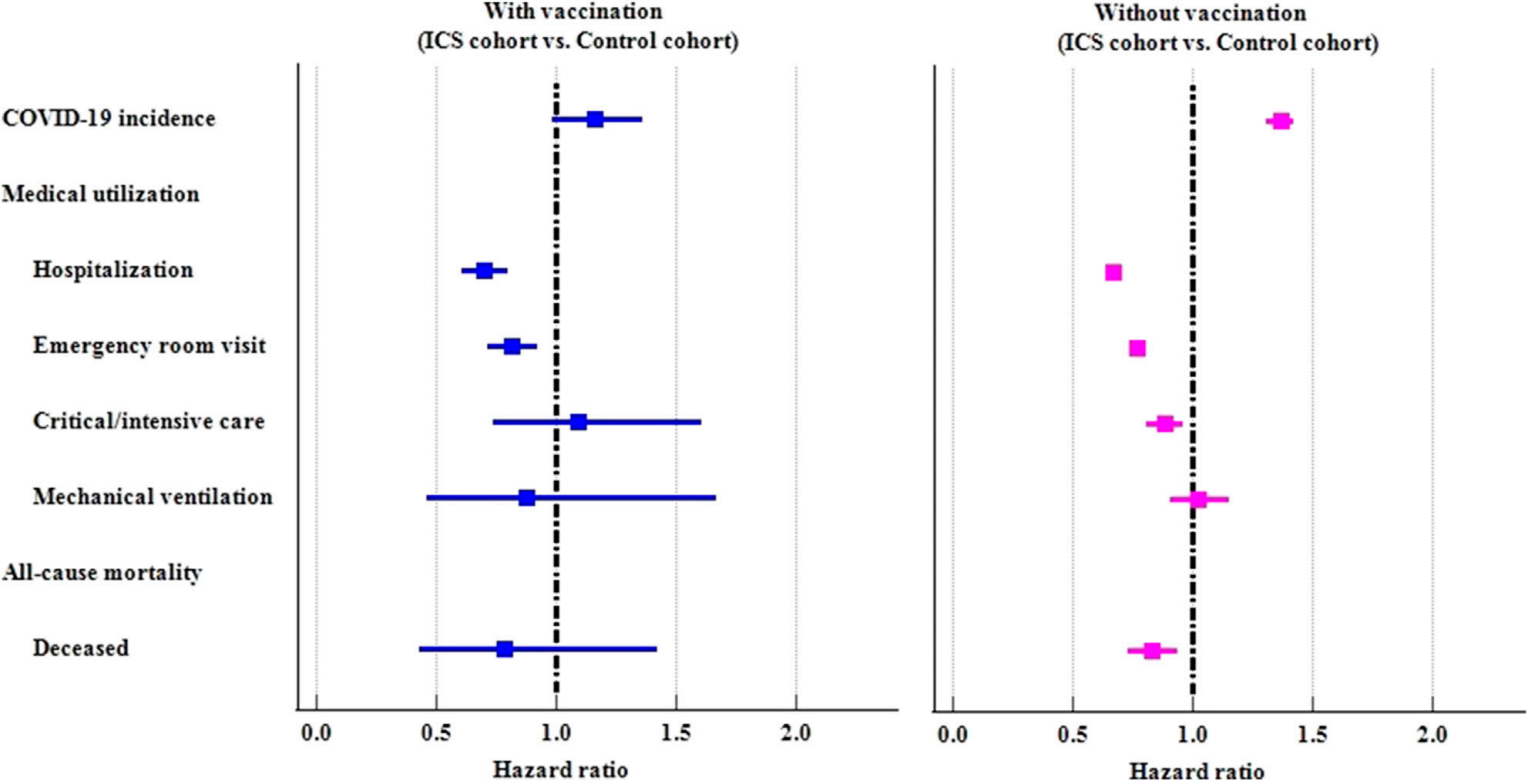
Forest plots of outcomes stratified by COVID-19 vaccination status.

We further examined the risk of outcomes in subgroups stratified by COVID-19 vaccination (Table 3; Figure 3). Among those who had ever received a COVID-19 vaccination, there were no statistically significant difference between ICS cohort and control cohort in COVID-19 incidence (HR: 1.161, 95%CI: 0.987–1.365). The ICS cohort had a lower risk of hospitalization (HR: 0.699, 95%CI: 0.609–0.803), and emergency department visits (HR: 0.814, 95%CI: 0.718–0.922) than the control cohort. Among those who had never received a COVID-19 vaccination, the ICS cohort exhibited a significantly higher risk of incidence of COVID-19 (HR: 1.366, 95%CI: 1.308–1.426), but a lower risk of medical utilization (HR: 0.667, 95%CI: 0.649–0.686, HR: 0.768, 95%CI: 0.747–0.789, HR: 0.881, 95%CI: 0.809–0.960 for hospitalization, emergency department visits, and critical care services, respectively). The ICS cohort also reveal lower risk of mortality (HR: 0.829, 95%CI:0.731–0.940) than control cohort among those subjects never vaccinated COVID-19 vaccines.

**TABLE 3:** Risk of outcomes _ stratified by COVID-19 vaccination.

Outcome (ICS vs. control cohort)	Adjusted hazard ratio (95% CI)^a^	
	With COVID-19 vaccination^b^ (n = 2,389)	Without COVID-19 vaccination^c^ (n = 55,118)
COVID-19 incidence	1.161 (0.987–1.365)	1.366 (1.308–1.426)*
Medical utilization		
Hospitalization	0.699 (0.609–0.803)	0.667 (0.649–0.686)
Emergency room visit	0.814 (0.718–0.922)	0.768 (0.747–0.789)*
Critical/intensive care	1.091 (0.741–1.606)	0.881 (0.809–0.960)
Mechanical ventilation	0.874 (0.458–1.669)	1.026 (0.911–1.155)
All-cause mortality		
Deceased	0.784 (0.432–1.425)	0.829 (0.731–0.940)

Note: ICS, inhaled corticosteroid; COVID, coronavirus disease; CI, confidence interval.

^a^ Data presented here are values in model 4, which were PSM, with age at index, sex, race; SES, lifestyles, comorbidities, vaccination, medication usage, and body mass index.

^b^ Vaccinated COVID-19, related vaccines (detail in Supplementary Table S2) on or before the index date.

^c^ Never vaccinated any COVID-19, related vaccines documented in their electronic medical records.

*proportionality <0.001.

#### Severity of disease

**FIGURE 4:**
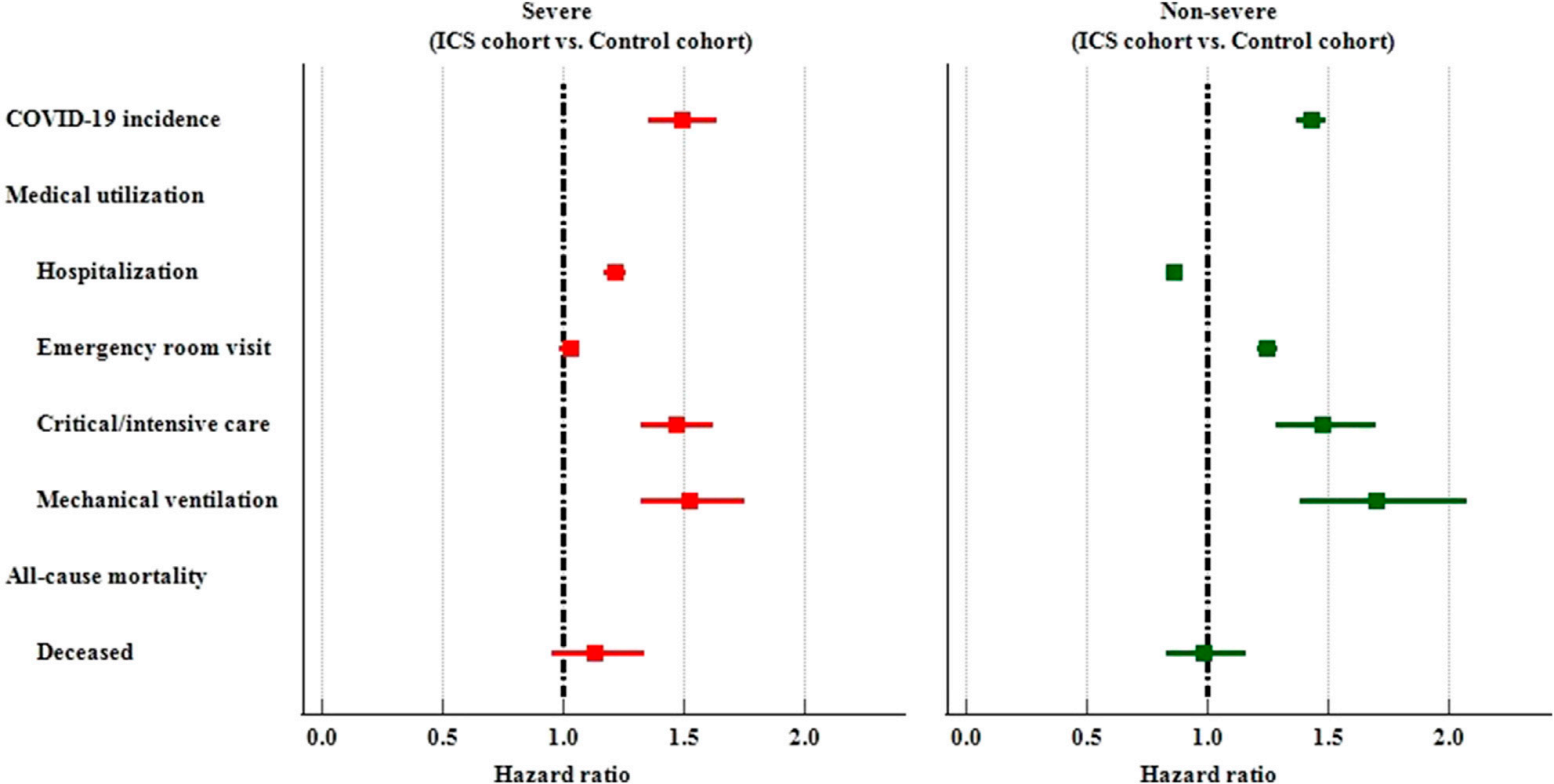
Forest plots of outcomes stratified by severe and non-severe.

We further divided study subjects into severe and non-severe based on whether they were hospitalized or visit emergency department or used critical care services within 1 month on or after the index date. Among the severe subjects, the ICS cohort had a higher risk of COVID-19 incidence (HR: 1.490, 95%CI: 1.353–1.640), hospitalization (HR:1.216, 95%CI:1.171–1.262), critical/intensive care (HR: 1.466, 95%CI: 1.326–1.622), and mechanical ventilation usage (HR: 1.522, 95%CI: 1.322–1.753) than the control cohort. Among thenon-severe subjects who had been prescribed an inhaled anti-inflammatory medication exhibited a significantly higher risk of incidence of COVID-19 (HR: 1.429, 95%CI: 1.368–1.492), and medical utilization (HR: 1.247, 1.477, 1.698 for emergency department visits, critical/intensive care, and mechanical ventilation, respectively). The non-severe ICS cohort also reveal lower risk of hospitalization (HR: 0.860, 95%CI:0.828–0.892) than non-severe control cohort. (Table 4; Figure 4).

**TABLE 4:** Risk of outcomes among ICS cohort compared to control cohort, stratified by inpatients and outpatients.

Outcome (ICS vs. control cohort)	Adjusted^a^ hazard ratio (95% CI)	
	Severe^b^ (n = 9,661)	Non-severe^c^ (n = 53,545)
COVID-19 incidence	1.490 (1.353–1.640)	1.429 (1.368–1.492)*
Medical utilization		
Hospitalization	1.216 (1.171–1.262)*	0.860 (0.828–0.892)*
Emergency room visit	1.027 (0.987–1.068)	1.247 (1.204–1.292)*
Critical/intensive care	1.466 (1.326–1.622)	1.477 (1.285–1.699)*
Mechanical ventilation	1.522 (1.322–1.753)	1.698 (1.387–2.077)*
All-cause mortality		
Deceased	1.131 (0.955–1.340)	0.981 (0.828–1.163)

Note: ICS, inhaled corticosteroid; COVID, coronavirus disease; CI, confidence interval.

^a^ Propensity score matching with age at index, sex, race, SES, lifestyles, comorbidities, vaccination, medication usage, and body mass index.

^b^ Hospitalized or visit emergency department or used critical care services within 1 month of asthma diagnosis on or after the index date.

^c^ Not hospitalized or visit emergency department or used critical care services within 1 month of asthma diagnosis on or after the index date.

*proportionality <0.001.

#### Sensitivity analyses

**FIGURE 5:**
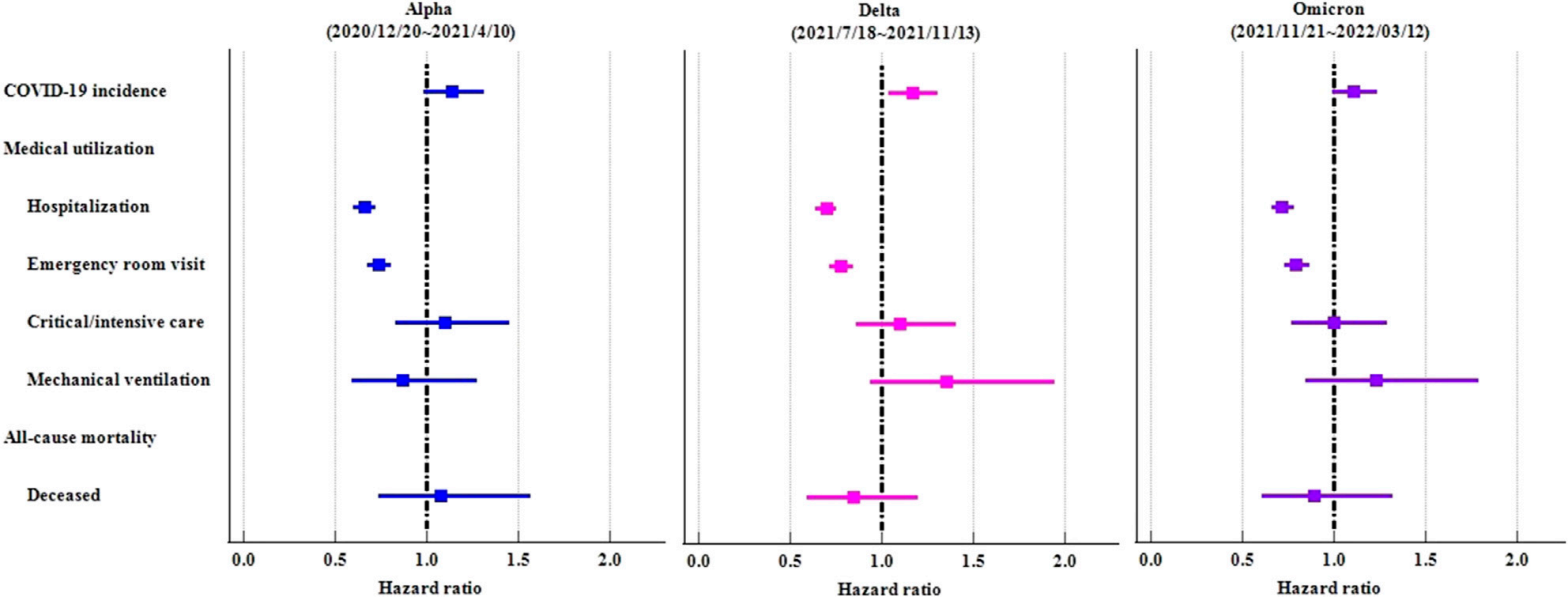
Forest plots of outcomes stratified by COVID-19 virus variants.

In order to demonstrate the consistency of results, we implemented the identical study design across various time waves of the COVID-19 virus (proxy Alpha: 2020/12/20∼2021/4/10; Delta: 2021/7/18∼2021/11/13; Omicron: 2021/11/21∼2022/3/12). Irrespective of viral prevalence, ICS cohort demonstrated elevated rates of COVID-19 in comparison to the control cohort. However, the statistical disparities were solely evident during the Delta variant pandemic (HR:1.167, 95%CI:1.042–1.307). Irrespective of viral prevalence, ICS cohort exhibited a significantly lower risk of hospitalization and emergency department visit (Table 5; Figure 5).

**TABLE 5:** Risk of outcomes among ICS cohort compared to control cohort, stratified by COVID-19 virus variants.

Outcome (ICS vs. control cohort)	Adjusted^a^ hazard ratio (95%CI)		
	Alpha^b^ (n = 4,097)	Delta^c^ (n = 5,161)	Omicron* (n = 4,756)
COVID-19 incidence	1.139 (0.986–1.316)	1.167 (1.042–1.307)	1.109 (0.991–1.240)
Medical utilization			
Hospitalization	0.659 (0.602–0.721)	0.697 (0.642–0.756)	0.718 (0.659–0.781)
Emergency room visit	0.740 (0.676–0.810)*	0.780 (0.717–0.848)*	0.796 (0.730–0.867)*
Critical/intensive care	1.099 (0.830–1.455)	1.102 (0.865–1.405)	0.998 (0.771–1.291)
Mechanical ventilation	0.870 (0.593–1.276)	1.350 (0.938–1.943)	1.230 (0.846–1.790)
All-cause mortality			
Deceased	1.076 (0.737–1.571)	0.846 (0.596–1.201)	0.895 (0.606–1.323)

Note: ICS, inhaled corticosteroid; COVID, coronavirus disease; CI, confidence interval.

^a^ Propensity score matching with age at index, sex, race, SES, lifestyles, comorbidities, vaccination, medication usage, and body mass index.

^b^ The study period was defined as 2020/12/20∼2021/4/10.

^c^ The study period was defined as 2021/7/18∼2021/11/13.

^d^ The study period was defined as 2021/11/21∼2022/3/12.

*proportionality <0.001.

## Discussion

This large retrospective cohort study utilized TriNetX, a real-world database, to investigate the association of ICS and the risk of COVID-19 incidence. In this analysis, we found that patients with newly diagnosed asthma who were prescribed with an inhaled corticosteroid had a higher risk of COVID-19 incidence than patients not on ICS. ICS usage lowered risks for medical utilization, including hospitalization, emergency department visits, and also the risk for mortality.

ICS is the backbone therapeutic option in the treatment of asthma. It not only controls the asthmatic condition, but it has also been suggested as a reliever in combination with formoterol in the current GINA guideline (GIf, 2022). Since the start of the COVID-19 pandemic outbreak, concerns have been raised regarding the use of ICS. Prior to the pandemic, there were studies showing that the use of ICS confers an increased risk of respiratory tract infection in asthma patients (Kim et al., 2019; Yang et al., 2019). Due to the lack of evidence that ICS increases the risk of getting COVID-19, many societies recommended continuing ICS (Hasan et al., 2020). In a recent study from Israel, the results suggested that recent and former use of ICS were not associated with increased risk of COVID-19 in 10,242 asthma patients, but asthma severity was not included in the data (Adir et al., 2022). The present study used a large number of patients and provides evidence that the use of ICS was related to an increased risk of COVID-19 acquisition.

It is recommended that asthma patients should be up-to-date with COVID vaccinations according to the major respiratory societies (Yang et al., 2019). In the subgroup analysis stratified by COVID vaccination, the increased risk of COVID-19 infection was lowered in the vaccination group. Based on the study results, COVID vaccination could mitigate the positive association between ICS use and the incidence of COVID infection. Although the risk of mortality was not significantly lower in patients with COVID vaccination, ICS did not increase the risk of mortality in patients without COVID vaccination. ICS use could lower risks of medical utilizations including hospitalization and emergency department visits with or without COVID vaccination. It is important to educate that asthma patients should keep their ICS and get COVID vaccination to prevent COVID infection.

Severity of asthma was difficult to measure and most studies performed assessments based on the patient’s medication use, according to the guidelines of the Global Initiatives for Asthma (GINA) (Yang et al., 2019). It was noted that severe asthma was associated with COVID-19-related death using the OpenSAFELY platform (Williamson et al., 2020). Schultze et al. reported high-dose ICS was associated with increased risks of COVID-19 related death in an analysis of 818,490 asthma patients in the United Kingdom, and the risk of confounding by indication was the major limitation (Schultze et al., 2020; Dolby et al., 2022). In the present study, we used medical utilizations including hospitalization or emergency department visit or used critical care services within 1 month to divide patients into severe and non-severe asthma. Using this operational definition to stratify asthma severity, we noticed that association between ICS use and the risk of COVID-19 incidence remained positive.

Hospitalization due to acute exacerbations is an important prognostic factor and exacerbation triggers are often unpredictable, such as viruses, pollens, pollution, and poor adherence (Convertino et al., 2020). Studies have shown that people with well-controlled asthma are not at increased risk of COVID-19-related death (Peters et al., 2020; Williamson et al., 2020). It is important to continue good asthma management to minimize the risks of asthma exacerbations, which decreased during the pandemic. The results of this study serve as a reminder that the use of ICS is possibly related to incident COVID-19. We recommend that patients with asthma under ICS use should also follow local health advice about hygiene strategies and use of personal protective equipment to prevent COVID infection and possible acute exacerbations following infections.

The mechanism linking ICS use and COVID-19 infection is not clear. It has been shown that a lower expression of ACE2 and TMPRSS2 in patients under ICS use could have a protective effect in preventing COVID-19 infection (Peters et al., 2020). Izquierdo et al. reported a significantly higher percentage of non-hospitalized patients using ICS following COVID-19 infection and concluded that ICS has a safe profile (Izquierdo et al., 2021). However, the study did not assess baseline asthma severity, and hospitalization following COVID-19 infection could have been a result of poor asthma control leading to acute exacerbation. These factors could have had an impact on the study outcome. Although there is still debate about the use of ICS and COVID-19 infectivity, our finding does not indicate that the use of ICS should be changed or avoided. In contrast, this finding contradicts the unfounded concerns related to the effects of ICS therapy in incident COVID-19, which may put asthmatic patients at real risk if they stop ICS treatment. Our study revealed a correlation between the use of ICS and reduced healthcare utilization rates as well as lower mortality rates.

Based on the findings of this article, it appears that we should place even greater emphasis on whether patients are attentive to practicing social distancing while using ICS. The use of ICS may indeed present an ideal opportunity for our mucous membranes to interact with the external environment. In the future, research should continue to investigate whether patients’ failure to maintain adequate social distancing during ICS use may be linked to COVID infection. If this hypothesis proves to be accurate, it becomes essential to educate patients about the significance of observing social distancing measures while using ICS.

This study had several strengths. We included newly diagnosed patients in our comparisons, rather than using existing asthma patients, which possibly excluded those with previous acute exacerbation histories, thereby potentially confounding the medical utilization results. We included laboratory data, including IgE level and BMI data, to help confirm the diagnosis and severity. The data contain both insured and uninsured patients with a large patient number, providing an accurate account of the burden of specific diagnoses on healthcare systems from the EMR.

However, there were some limitations in this study. First, we may have underestimated the risk of COVID infection if patients did not seek medical help when symptoms developed under ICS use. Regional variation existed in reporting COVID symptoms during the pandemic (Abutiban et al., 2022). Second, the exact risk could not be estimated if patients were lost to follow-up in a single medical institution. The exact ICS dose was not ascertained which could represent severity of asthma and the number of vaccination doses was not taken into consideration in this study. Third, we did not include all of the type 2 inflammation markers, such as eNO and all of the IgE laboratory results, which vary according to the method used, with different reference values. Fourth, we were not able to set index date of medication as matching covariate, which could lead to potential immortal time bias. Fifth, scleroderma is a distinct type of rheumatic disease could affect the smooth muscles as the respiratory tract and could potentially influence the outcome evaluation. However, we concentrate on patients with asthma, so we will include this disease in the limitations section. We hope that subsequent studies will be able to incorporate this disease. Lastly, we might have underestimated the risk by excluding patients with COVID vaccinations and we did not further explore the impact of ICS use on COVID-19-related death. Further studies are warranted.

## Conclusion

We observed a positive association between the use of ICS and incident COVID within a subsequent year of the initial diagnosis of asthma, based on a comparison with propensity score-matched patients not exposed to ICS. Patients on ICS should follow their treating physician’s advice to prevent COVID-19 infection.

## Data Availability

The data analyzed in this study is subject to the following licenses/restrictions: Datasets analyzed in the present study can be provided by the authors upon reasonable request. Requests to access these datasets should be directed to https://trinetx.com/.
