# Supplementary material for: Associations between COVID-19 outcomes and asthmatic patients with inhaled corticosteroid
Source: Front Pharmacol. 2023 Nov 1;14:1204297. doi: 10.3389/fphar.2023.1204297 (PMC10646298; doi:10.3389/fphar.2023.1204297)
Supplement: Supplementary file 1 [file DataSheet1.PDF]

Supplementary material

Table S1. SARS coronavirus 2 related RNA laboratory tests used in the present study

Codes	Description
TNX:9088	SARS coronavirus 2 and related RNA [Presence]
UMLS:LNC:94306-8	SARS-CoV-2 (COVID-19) RNA panel - Specimen by NAA with probe detection
UMLS:LNC:41458-1	SARS coronavirus RNA [Presence] in Specimen by NAA with probe detection
UMLS:LNC:94531-1	SARS-CoV-2 (COVID-19) RNA panel - Respiratory specimen by NAA with probe detection
UMLS:LNC:94764-8	SARS-CoV-2 (COVID-19) whole genome [Nucleotide sequence] in Isolate or Specimen by Sequencing
UMLS:LNC:94511-3	SARS-CoV-2 (COVID-19) ORF1ab region [Cycle Threshold #] in Specimen by NAA with probe detection

Note:
TNX, TriNetX curated
UMLS, Unified Medical Language System
LNC, Logical Observation Identifiers Names and Codes (LOINC)

Table S2. COVID-19 related vaccination code

Dimension	Codes	Description
procedure	UMLS:CPT:91300	Severe acute respiratory syndrome coronavirus 2 (SARS-CoV-2) (coronavirus disease [COVID-19]) vaccine, mRNA-LNP, spike protein, preservative free, 30 mcg/0.3 mL dosage, diluent reconstituted, for intramuscular use
procedure	UMLS:CPT:91301	Severe acute respiratory syndrome coronavirus 2 (SARS-CoV-2) (coronavirus disease [COVID-19]) vaccine, mRNA-LNP, spike protein, preservative free, 100 mcg/0.5 mL dosage, for intramuscular use
procedure	UMLS:CPT:91302	Severe acute respiratory syndrome coronavirus 2 (SARS-CoV-2) (coronavirus disease [COVID-19]) vaccine, DNA, spike protein, chimpanzee adenovirus Oxford 1 (ChAdOx1) vector, preservative free, 5x1010 viral particles/0.5 mL dosage, for intramuscular use
procedure	UMLS:CPT:91303	Severe acute respiratory syndrome coronavirus 2 (SARS-CoV-2) (coronavirus disease [COVID-19]) vaccine, DNA, spike protein, adenovirus type 26 (Ad26) vector, preservative free, 5x1010 viral particles/0.5 mL dosage, for intramuscular use
procedure	UMLS:CPT:91304	Severe acute respiratory syndrome coronavirus 2 (SARS-CoV-2) (coronavirus disease [COVID-19]) vaccine, recombinant spike protein nanoparticle, saponin-based adjuvant, preservative free, 5 mcg/0.5 mL dosage, for intramuscular use
procedure	UMLS:CPT:91305	Severe acute respiratory syndrome

		coronavirus 2 (SARS-CoV-2) (coronavirus disease [COVID-19]) vaccine, mRNA-LNP, spike protein, preservative free, 30 mcg/0.3 mL dosage, tris-sucrose formulation, for intramuscular use
procedure	UMLS:CPT:91306	Severe acute respiratory syndrome coronavirus 2 (SARS-CoV-2) (coronavirus disease [COVID-19]) vaccine, mRNA-LNP, spike protein, preservative free, 50 mcg/0.25 mL dosage, for intramuscular use
procedure	UMLS:CPT:91307	Severe acute respiratory syndrome coronavirus 2 (SARS-CoV-2) (coronavirus disease [COVID-19]) vaccine, mRNA-LNP, spike protein, preservative free, 10 mcg/0.2 mL dosage, diluent reconstituted, tris-sucrose formulation, for intramuscular use
procedure	UMLS:CPT:91308	Severe acute respiratory syndrome coronavirus 2 (SARS-CoV-2) (coronavirus disease [COVID-19]) vaccine, mRNA-LNP, spike protein, preservative free, 3 mcg/0.2 mL dosage, diluent reconstituted, tris-sucrose formulation, for intramuscular use
procedure	UMLS:CPT:91309	Severe acute respiratory syndrome coronavirus 2 (SARS-CoV-2) (coronavirus disease [COVID-19]) vaccine, mRNA-LNP, spike protein, preservative free, 50 mcg/0.5 mL dosage, for intramuscular use
procedure	UMLS:CPT:91311	Severe acute respiratory syndrome coronavirus 2 (SARS-CoV-2) (coronavirus disease [COVID-19]) vaccine, mRNA-LNP, spike protein, preservative free, 25 mcg/0.25 mL dosage, for intramuscular use

procedure	UMLS:CPT:91312	Severe acute respiratory syndrome coronavirus 2 (SARS-CoV-2) (coronavirus disease [COVID-19]) vaccine, mRNA-LNP, bivalent spike protein, preservative free, 30 mcg/0.3 mL dosage, tris-sucrose formulation, for intramuscular use
procedure	UMLS:CPT:91313	Severe acute respiratory syndrome coronavirus 2 (SARS-CoV-2) (coronavirus disease [COVID-19]) vaccine, mRNA-LNP, spike protein, bivalent, preservative free, 50 mcg/0.5 mL dosage, for intramuscular use
procedure	UMLS:CPT:91314	Severe acute respiratory syndrome coronavirus 2 (SARS-CoV-2) (coronavirus disease [COVID-19]) vaccine, mRNA-LNP, spike protein, bivalent, preservative free, 25 mcg/0.25 mL dosage, for intramuscular use
procedure	UMLS:CPT:91315	Severe acute respiratory syndrome coronavirus 2 (SARS-CoV-2) (coronavirus disease [COVID-19]) vaccine, mRNA-LNP, bivalent spike protein, preservative free, 10 mcg/0.2 mL dosage, diluent reconstituted, tris-sucrose formulation, for intramuscular use
procedure	UMLS:CPT:91316	Severe acute respiratory syndrome coronavirus 2 (SARS-CoV-2) (coronavirus disease [COVID-19]) vaccine, mRNA-LNP, spike protein, bivalent, preservative free, 10 mcg/0.2 mL dosage, for intramuscular use
procedure	UMLS:CPT:91317	Severe acute respiratory syndrome coronavirus 2 (SARS-CoV-2) (coronavirus disease [COVID-19]) vaccine, mRNA-LNP, bivalent spike protein, preservative free, 3 mcg/0.2 mL dosage, diluent reconstituted, tris-sucrose

		formulation, for intramuscular use
procedure	UMLS:ICD10PCS: XW013S6	Introduction of COVID-19 Vaccine Dose 1 into Subcutaneous Tissue, Percutaneous Approach, New Technology Group 6
procedure	UMLS:ICD10PCS: XW013T6	Introduction of COVID-19 Vaccine Dose 2 into Subcutaneous Tissue, Percutaneous Approach, New Technology Group 6
procedure	UMLS:ICD10PCS: XW013U6	Introduction of COVID-19 Vaccine into Subcutaneous Tissue, Percutaneous Approach, New Technology Group 6
procedure	UMLS:ICD10PCS: XW013V7	Introduction of COVID-19 Vaccine Dose 3 into Subcutaneous Tissue, Percutaneous Approach, New Technology Group 7
procedure	UMLS:ICD10PCS: XW013W7	Introduction of COVID-19 Vaccine Booster into Subcutaneous Tissue, Percutaneous Approach, New Technology Group 7
procedure	UMLS:ICD10PCS: XW023S6	Introduction of COVID-19 Vaccine Dose 1 into Muscle, Percutaneous Approach, New Technology Group 6
procedure	UMLS:ICD10PCS: XW023T6	Introduction of COVID-19 Vaccine Dose 2 into Muscle, Percutaneous Approach, New Technology Group 6
procedure	UMLS:ICD10PCS: XW023U6	Introduction of COVID-19 Vaccine into Muscle, Percutaneous Approach, New Technology Group 6
procedure	UMLS:ICD10PCS: XW023V7	Introduction of COVID-19 Vaccine Dose 3 into Muscle, Percutaneous Approach, New Technology Group 7
procedure	UMLS:ICD10PCS: XW023W7	Introduction of COVID-19 Vaccine Booster into Muscle, Percutaneous Approach, New Technology Group 7
medication	NLM:CVX:213	SARS-CoV-2 (COVID-19) Vaccine

Note:
UMLS, Unified Medical Language System.
CPT, Current Procedural Terminology.
ICD10PCS, ICD-10 Procedure Coding System.
NLM, National Library of Medicine.
CVX, Vaccines Administered.

Table S3. Risk of outcomes (different follow up periods)

Outcome (ICS vs. control cohort)	Adjusted[a] hazard ratio (95% CI)				
	**1 to 30 days**	**1 to 90 days**	**90 to 365 days**	**180 to 365 days**	**270 to 365 days**
COVID-19 incidence	**1.803 (1.637-1.986)**	**1.624 (1.520-1.735)**	**1.220 (1.162-1.281)**	**1.213 (1.141-1.290)**	**1.188 (1.086-1.298)**
Medical utilization					
Hospitalization	**0.699 (0.674-0.724)***	**0.681 (0.660-0.702)***	**0.648 (0.612-0.686)***	**0.669 (0.619-0.724)***	**0.844 (0.740-0.963)**
Emergency room visit	**0.598 (0.571-0.627)***	**0.688 (0.665-0.713)***	**1.063 (1.004-1.126)**	1.034 (0.960-1.115)	1.102 (0.983-1.237)
Critical/intensive care	0.915 (0.815-1.026)	0.928 (0.842-1.024)	1.013 (0.874-1.174)	1.030 (0.847-1.252)	1.146 (0.851-1.543)
Mechanical ventilation	1.053 (0.903-1.227)	1.034 (0.905-1.182)	1.113 (0.898-1.380)	1.230 (0.912-1.658)	1.077 (0.689-1.684)
All-cause mortality					
Deceased	0.971 (0.745-1.265)	0.843 (0.701-1.014)	**0.833 (0.713-0.972)**	0.875 (0.717-1.069)	0.810 (0.599-1.095)

Note:
ICS: inhaled corticosteroid, COVID: Coronavirus Disease, CI: Confidence interval
a. Propensity score matching was performed on age at index, sex, race, social economic status, lifestyles, vaccination, comorbidities, medication usage, and body mass index.
* proportionality < 0.001
